# RecF protein targeting to postreplication (daughter strand) gaps I: DNA binding by RecF and RecFR

**DOI:** 10.1093/nar/gkad311

**Published:** 2023-05-01

**Authors:** Camille Henry, Neema Mbele, Michael M Cox

**Affiliations:** Department of Biochemistry, University of Wisconsin-Madison, Madison, WI 53706-1544, USA; Department of Biochemistry, University of Wisconsin-Madison, Madison, WI 53706-1544, USA; Department of Biochemistry, University of Wisconsin-Madison, Madison, WI 53706-1544, USA

## Abstract

In bacteria, the repair of post-replication gaps by homologous recombination requires the action of the recombination mediator proteins RecF, RecO and RecR. Whereas the role of the RecOR proteins to displace the single strand binding protein (SSB) and facilitate RecA loading is clear, how RecF mediates targeting of the system to appropriate sites remains enigmatic. The most prominent hypothesis relies on specific RecF binding to gap ends. To test this idea, we present a detailed examination of RecF and RecFR binding to more than 40 DNA substrates of varying length and structure. Neither RecF nor the RecFR complex exhibited specific DNA binding that can explain the targeting of RecF(R) to post-replication gaps. RecF(R) bound to dsDNA and ssDNA of sufficient length with similar facility. DNA binding was highly ATP-dependent. Most measured *K*_d_ values fell into a range of 60–180 nM. The addition of ssDNA extensions on duplex substrates to mimic gap ends or CPD lesions produces only subtle increases or decreases in RecF(R) affinity. Significant RecFR binding cooperativity was evident with many DNA substrates. The results indicate that RecF or RecFR targeting to post-replication gaps must rely on factors not yet identified, perhaps involving interactions with additional proteins.

## Graphical Abstract

**Graphical Abstract:**
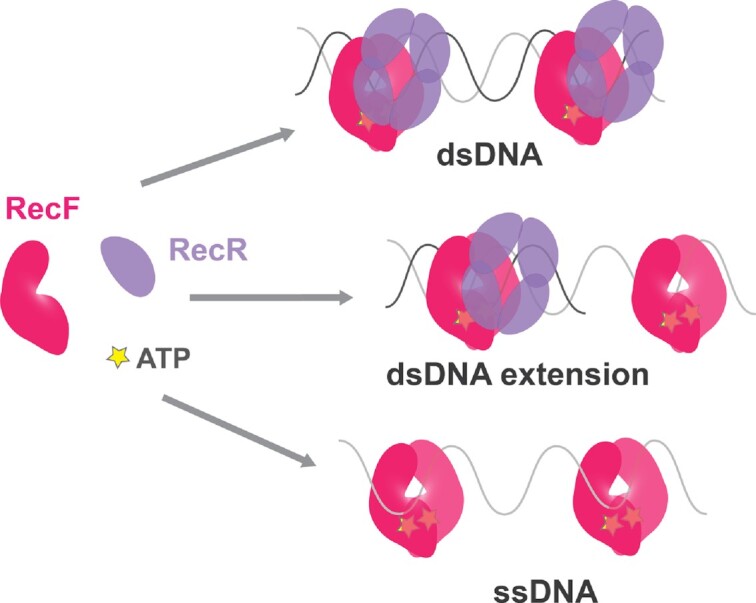
RecF or RecFR bind to all DNA structures with low nM affinity, with no specificity for gap ends.

## INTRODUCTION

Cells from bacteria to human are constantly exposed to various stresses that can cause DNA damage, which can lead to genetic disorders, genomic instability, and even cell death (1–5). When replisomes encounter DNA lesions in the template, they can stall, collapse, or undergo lesion-skipping. Lesion-skipping uniquely allows the replisome to progress unimpeded. However, it leaves behind a lesion-containing post-replication gap. Size estimates suggest these gaps appear to be in the range of 100–800 nucleotides in length (6) but may be enlarged by the RecJ nuclease (7,8). In *Escherichia coli*, the generation of post-replication gaps occurs up to several times each replication cycle (6). In this process, the replisome disengages at the site of the lesion and re-initiates downstream (9). As with all single strand gaps, post-replication gaps are presumably coated with single-stranded DNA binding protein or SSB, protecting the ssDNA and recruiting SSB interaction partners including RecO, RecQ, RecJ, RecG, RadD, ExoI, HolC (χ), PolII, PriA, RarA and PriC (10–12).

A lesion-containing ssDNA gap behind the replication fork imposes some special repair challenges. In nucleotide or base excision repair, lesions are simply cleaved out of the damaged strand, and repair is directed by the undamaged complementary strand. When the lesion is in a single strand, as is the case in a post-replication gap, there is no paired and undamaged strand to repair against. The lesion cannot be cleaved out of the single strand gap, as this would create a double strand break. Double strand break repair is efficient (albeit expensive). However, in this case, double strand break repair at a location behind the active replication fork would lead to creation of an unscheduled replication fork and over-replication of a segment of the genome. Most repair occurs via a recombination pathway known by many names, including post-replication gap repair, daughter strand gap repair and single strand gap (SSG) repair (13,14). Post-replication gap repair creates a transient crossover that links the products of replication behind the fork. Failure to process this intermediate can block cell division and lead to cell death (6,7,15,16). Failure to initiate gap repair leaves a template discontinuity that will create a double strand break when the next replisome arrives. It is thus essential that post-replication gap repair proceed rapidly and efficiently while keeping the lesion-containing ssDNA intact.

Post-replication gap repair is observed from bacteria to humans and requires the action of recombination mediator proteins (RMPs). RMPs are the product of a convergent evolution and therefore present different molecular mechanisms (13,17). Nonetheless, all RMPs share a common function, they help bypass the physical barrier represented by single-stranded DNA binding proteins tightly bound to ssDNA and facilitate the loading of the RecA/Rad51 recombinases. RMPs participate in the error free repair of the lesion via homologous recombination. To function efficiently in post-replication gap repair, RMPs must be precisely targeted to lesion-containing post-replication gaps. Targeting to other gaps, such as the many lagging strand gaps created during replication, would waste resources and could possibly be deleterious.

In bacteria, the RMP function is carried out primarily by the RecF, RecO and RecR proteins (13,18–26). These three bacterial RMPs comprise the RecF epistasis group of proteins based on the very similar phenotypes of deletion mutants with respect to effects on UV or mitomycin C resistance, conjugational recombination, and more. These proteins define the major path of post-replication gap repair in bacteria that is often called the RecFOR pathway. The entire RecFOR pathway relies on contributions from many other proteins including RecA, RecQ, RecJ, RecG, RuvAB, RarA and others (13,18–21,26–28).

In spite of the clear genetic relationship seen between *recF*, *recO* and *recR*, no complex containing RecF, RecO and RecR altogether has been isolated to date (29,30). Two distinct binary complexes, RecOR and RecFR, have been well-documented (22,23,29,31–33). RecF and RecO compete for the binding to the TOPRIM domain of RecR with a preferential binding to RecF (29,33). As the epistasis observed between *recF*, *recO* and *recR* have been further explored, phenotypic differences have emerged, particularly between *recO* and *recF* (34–39). DNA compaction following UV irradiation requires RecO protein, while RecF appears to have only a partial effect (34). RecF protein but not RecO often colocalizes with the replisome (36). RecF and RecO are thus spatiotemporally separated *in vivo* (36). Additionally, while all proteins are needed for proper SOS induction, only RecF is required for PolV mutagenesis (37). Altogether, this suggests that RecFR and RecOR may have distinct functions within the RecFOR system. When using the term RecFOR, we are therefore referring to a pathway or system, not to a stable protein complex.

There are two distinct functions of the RecFOR system. First, the appropriate proteins must be targeted to lesion-containing post-replication gaps (while avoiding gaps that do not require repair). Second, they must facilitate the loading of RecA protein into that gap. The RecOR complex harbors an evident loading function, as RecOR is necessary and sufficient to facilitate the loading of RecA onto SSB coated ssDNA *in vitro* (13,17,22–24,33,40). The loading mechanism involves allosteric modulation of RecO created upon interaction with SSB. This modulates the cooperativity of RecO binding to RecR, shifting the equilibrium between RecO_2_R_4_ and RecOR_4_ toward the latter. This leaves a space available for RecA loading (41). However, the role of RecF as RMP is less clear. Neither RecF nor RecFR will load RecA onto SSB-coated ssDNA on their own, which would suggest a role of RecF in the targeting function. RecF inhibits the function of RecO protein under most experimental conditions (23,33,42). RecF enhances the RecA nucleofilament formation in the presence of RecOR under very specific conditions *in vitro* (23,24,43), primarily when the interaction of RecO with either SSB (23) or RecR is constrained (23,24,43).

The mechanism by which the RecFOR system is targeted to post-replication gaps has not been defined. When RecF binding to DNA is limited to short duplexes flanking SSB-coated single-stranded DNA, a RecF-mediated enhancement of RecOR loading of RecA can be observed (23,24). This has led to a model in which RecF and/or RecFR binds specifically to gap ends and facilitates the action of RecOR, thus loading RecA into the targeted gaps (14,15). Specific binding of RecF and/or RecFR to gap ends is, to date, the most prominent hypothesis put forward to explain the targeting of the RecFOR system to post-replication gaps. However, in these studies, the substrates used were long ssDNA fully coated with SSB flanked with very short duplexes. The combinations effectively prevents RecF binding to any location away from gap ends. A specific binding of RecF or RecFR to gap ends has not been evident in other studies (23,31,32). Electron microscopy coupled to gold labeling shows RecF randomly bound along the entire dsDNA region (44). Further studies revealed that RecF can prevent RecA filaments from extending into duplex regions adjacent to a gap (44,45). Notably, confinement of RecA to the gap occurred only if sufficient RecF was present *in vitro* to entirely coat the duplex region (44,46).

A gap-targeting mechanism based on RecF(R) binding to gap ends is problematic for an important additional reason. Lesion-containing post-replication gaps requiring RecA-mediated post-replication gap repair occur in a milieu in which they likely represent just a small fraction of the gaps that appear during replication. If the RecF(R) proteins bound to gap ends and directed the RecFOR system to all gaps, the resulting RecA filaments and their potential for the creation of joint molecules linking the sister chromosomes behind the fork could result in genomic chaos. The RecFOR system must be targeted not just to gaps but to the small number of post-replication gaps in which lesions are present. In addition, the activity of the RecOR complex, which can load RecA onto SSB-coated ssDNA at any location, must be constrained so that it operates primarily in appropriate gaps.

RecF is a member of the ATP binding cassette (ABC)-ATPase clade and is similar to structural homologs: Rad50 and structure maintenance chromosome (SMC) proteins, (47,48). Despite a diversity of functions, all ABC systems share structural similarities and key steps in their hydrolytic cycle mechanism (47,49). Their main characteristics are (i) the presence of Walker A, Walker B, and signature features and (ii) the lack of the arginine finger found in the other ATPases. The lack of an arginine finger imposes a head-to-head arrangement within the dimers, in which two ATP molecules are located at the interfaces created between the Walker A and signature domain of the opposite monomers. In the case of the ABC systems, the conformational change resulting from the cooperativity between ATP binding and dimerization is proposed to itself transduce the motion to the molecule partners while the ATP-hydrolysis is used to recycle the systems (47,49,50). RecF and structural homologs have often been grouped with proteins not involved in transport, such as SufC and DNA repair proteins (MutS, Uup, UvrA, SbcC, MukB, RecN). Nonetheless, a comprehensive classification revealed that ABC ATPases are ancient systems that started to structurally diverge pre-LUCA (47). Thus, ABC structural feature evolution may provide a more relevant classification. This would group RecF with ancestral proteins such as RecN and proteins that contain a coiled coil but lack the hinge present in SMC and MukB and the Zn hook present in Rad50 and SbcC. *In vitro*, RecF binds both ss and dsDNA with an apparent preference for dsDNA in the presence of ATP (32,51–53). Interaction with its RecR partner stabilizes RecF binding to dsDNA (31,32).

The precise targeting of the RecFOR system to lesion-containing post-replication gaps represents a significant unresolved problem in the ongoing effort to understand bacterial DNA metabolism. In spite of issues cited above, binding of RecF to gap ends is still the prevailing paradigm in discussions of RecFOR system targeting to post-replication gaps (7,12,13,23,24,27,28,32,36,41,43,44,53–61). To thoroughly test that targeting hypothesis and to help guide new research on RecFOR system targeting in post-replication gap repair, we now present a comprehensive analysis of RecF and RecFR binding to a wide range of DNA structures. The results reveal some new properties of the RecF(R) interaction with DNA. However, they provide no support for a post-replication gap targeting mechanism that involves specific RecF or RecFR binding to gap ends.

## MATERIALS AND METHODS

### Reagents

Reagents used for protein purification and in the reaction buffer were purchased from Fisher Scientific, Sigma, Merck-Millipore or GoldBio. Dithiothreitol (DTT) and Adenosine triphosphate pH 7 (ATP) stock were prepared using ultrapure water, filter sterilized, aliquoted, and flash frozen. For each experiment freshly melted aliquots were used.

### Cloning

The *E. coli recR* gene was cloned into a dual-tag purification system. This system allows the purification of a protein of interest in two steps. The first step of purification is a maltose binding protein affinity purification. This is followed by cleavage using the SUMO protease Ulp1 between the MalE-6His-Smt3 and RecR. Then a second His purification is used to separate the cleaved MalE-6His and SUMO protease from RecR. Briefly, the recR gene was amplified and cloned into the pJOE4905.1 vector using the SfoI/HindIII restriction enzymes. The *recR* amplification was realized with the following primers: recR’start 5′-ATGCAGACCAGCCCGCTG and recRdsHindIII 5′-GGATATCAAGCTTTTAAAAACGAATCTTATGACGCCC to generate the pCJH0006 plasmid.

### Proteins

RecF was purified as described earlier (31) with the following modifications. The Phosphocellulose P11 column was replaced by a Cellulose Phosphate (C2258 from Sigma) column, followed by the classic chromatography step on PBE94. The PBE94 fractions containing RecF were pooled and concentrated with 10K Amicon ultra (Merck-Millipore) and dialyzed against 1xM buffer (20 mM Tris–Cl 80%+ pH 7.5, 0.1 mM EDTA + 2 mM DTT) containing 1 M NaCl and 10% glycerol, then loaded onto a gel filtration column Sephacryl S200 (GE healthcare-Cytivia) in the same buffer. Fractions containing the purified RecF (monomeric) were pooled and dialyzed against the RecF storage buffer (1xM + 100 mM NaCl + 60% glycerol + 2 mM DTT).

The *E. coli* RecR protein was purified using a dual-tag purification method allowing the purification of a protein of interest by a first step of maltose binding protein affinity purification, followed by the cleavage by the SUMO protease Ulp1 between the MalE-6His-Smt3 and the protein (62). Thus leaving the cleaved RecR protein without any tag. Briefly, the MalE-6His-Smt3 and RecR was expressed from 4 l of STL2669 culture transformed by pJOE-*recR* allowing the expression of the fusion protein under the control of a Rhamnose promoter. At OD_600_: 0.4 the expression was induced by adding 0.2% Rhamnose. Cells were grown overnight at 37°C and then harvested by centrifugation. The cell pellet of 19.73 g was flash frozen with the liquid nitrogen. Cell pellet was resuspended in 20% (w/v) of tris-sucrose solution. After resuspension, cells were lysed by sonication. The cell extract was clarified by centrifugation and the supernatant was dialyzed against R buffer containing 100 mM NaCl and 10% glycerol. We carried out a first step of HisTag purification in order to get cleaner lysate on the amylose column. The fractions containing the His tagged protein were pooled and dialyzed against R buffer containing 100 mM NaCl and 10% glycerol. The dialysed pool was loaded onto a gravity column of amylose resin pre-equilibrated with loading solution, after a wash of 5 column volumes, the protein was eluted with loading buffer supplemented with 20 mM maltose into the initial buffer. Fractions containing the fusion protein were pooled. The Ulp1 protease was added to the pooled fraction at the ratio 1:100 and the fusion protein was digested overnight at 4°C. The digestion product was dialyzed against R buffer containing 100 mM NaCl and 10% glycerol. Finally, RecR was separated from the cleaved MalE-6His-Smt3 and un-cleaved product by His purification. Cleaved RecR was eluted in the flow through.

After purification, the concentration of RecF and RecR were determined using their respective extinction coefficients ϵ_280_: 3.87 × 10^4^ M^−1^ cm^−1^ (63) and ϵ_280_: 5.6 × 10^3^ M^−1^ cm^−1^ (45). The purified proteins were free of nuclease activities and the protein sequences and purity were confirmed by Mass spectrometry at the Biotechnology Center UW-Madison.

### DNA substrates

DNA substrates used in this study are listed in Tables 1 and 2. The oligonucleotides utilized to generate the different DNA substrates are listed in the following table. Oligonucleotides were designed to avoid unwanted secondary structure and purchased from Integrated DNA Technologies or Genelink (for Thymine dimer oligo). When indicated a fluorescein (FAM) was added as specified either internally, or at the 5′ or 3′ end of the DNA.

**Table 1.** Binding characteristics of Hill-slope fits obtained for ssDNA, dsDNA and double stranded DNA with a ssDNA extension. Indicated in the table: the type of DNA, the schematic of the DNA substrates for which the FAM label is represented by a green star and the complementary DNA is indicated in light grey, the presence or not of ATP and RecR, the Bmax values with the higher value of the 95% confidence interval, the *K*_d_ with the higher value of the 95% confidence interval, the cooperativity value (*h*) and the goodness of the fit (*r*^2^)

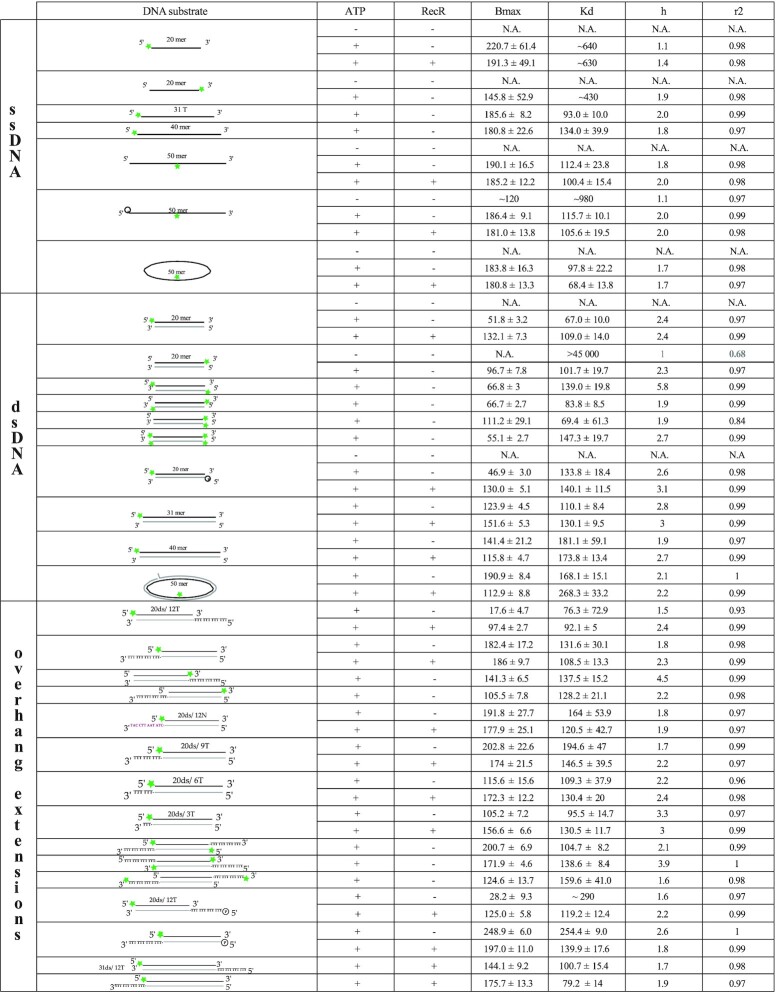

**Table 2.** Binding characteristics of Hill-slope fits obtained for DNAs containing CPD or single strand nick or gap region. Indicated in the table: the type of DNA, the schematic of the DNA substrates for which the FAM label is represented by a green star, the CPD lesion by a red triangle and the complementary DNA is indicated in light grey, the presence or not of ATP and RecR, the Bmax values with the higher value of the 95% confidence interval, the *K*_d_ with the higher value of the 95% confidence interval, the cooperativity value (*h*) and the goodness of the fit (*r*^2^)

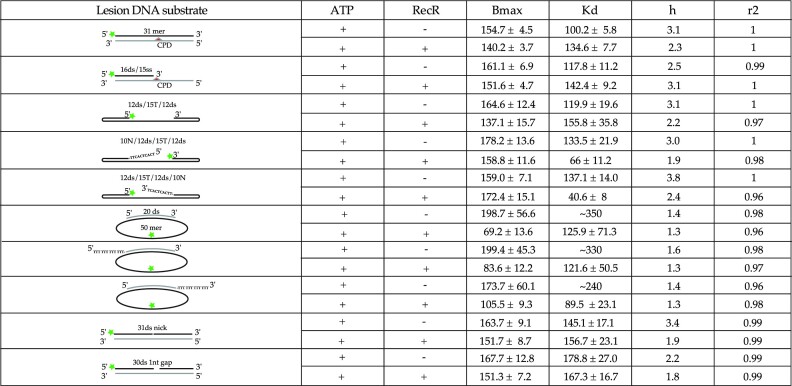

Duplex DNA was generated by annealing oligonucleotides harboring a complementary region, with at least one FAM labeled for the DNA used in the fluorescence anisotropy assay. ‘Gap’ DNAs with hairpins at each end were folded using the same annealing protocol. A single FAM labeled oligonucleotide containing two hairpin regions separated by a string of 15T was used to create the gap region in these substrates. Annealing reactions were carried out in 40 mM Tris–Cl pH 7.5, 20 mM MgCl_2_ and 80 mM NaCl buffer. The reactions were heated at 98°C for 10 min with a thermocycler and then cooled down to 4°C through a linear gradient of 1°C Celsius every minute. After annealing, the cooled reactions were loaded on 1xTris borate EDTA (TBE) 12 or 15% acrylamide gel (native PAGE) and submitted to electrophoresis. The annealed substrates were then gel PAGE purified.

The iFAM oligonucleotides (consisting of two regions of 10 nucleotides flanking 30 thymidylate residues, in which the two flanking regions are complementary to the 20mer m13) was used to generate: (i) the dephosphorylated 50mer, (ii) the circular gap DNA and (iii) after further modifications the circular ssDNA and the circular dsDNA. The dephosphorylated DNA was obtained by treating 200 μl of 2 μM iFAM 50mer (5Phospho) with 1 μl of Alkaline Phosphatase (New England Biolabs) for 2 h at 37 °C, followed by phenol/chloroform extraction and ethanol precipitation. The circular gap DNAs, the circular ssDNA and the circular double stranded DNA all started with a similar annealing step with the adequate complementary DNA (20mer with or without ssDNA extensions, or with the 5′ Phosphorylated 20mer for the first step of cds construction). The annealing reactions were carried out as described above with a final concentration of probes limited to a maximum of 2 μM in molecules (a substantial decrease in annealing efficiency was observed at higher concentration). After annealing, reactions were ligated using the T4 DNA Ligase (Themofisher) for 15 min at room temperature then kept overnight at 4°C. After annealing, the circular gap DNAs were loaded on 12% or 15% native TBE polyacrylamide gel and the annealed DNAs were PAGE purified. The css DNA was obtained by treating the PAGE purified annealed gap DNA (ligated) with the DNA Polymerase PolI (New England Biolabs). The proofreading activity of PolI was used to remove the complementary 20mer oligonucleotide that was used to guide ligation. Finally, in the case of the iFAM cds, the circular gap DNA ligated, obtained by annealing the iFAM to the 20mer m13 5′ Phosphorylated, was directly purified by phenol/chloroform extraction and ethanol precipitation. Then the gap DNA (5′Phospho) was used as template in a primer extension reaction using the Klenow fragment of PolI (lacking the proofreading activity, New England Biolabs). The polymerization reaction was carried out in presence of 50 μM of dATP for each μM of DNA template, to fill the gap (consisting of 30 thymidylate residues). The polymerized cds DNA was then purified by Phenol/Chloroform extraction and ethanol precipitation and the purified DNA was ligated as described above. Another purification was conducted before loading the DNA on a gel followed by a PAGE purification. The proper circularization (ligation) of the DNA was verified by (resistance to) ExoI (ssDNA) using the assay recommended by NEB. The final DNA concentrations were measured with NanoDropOne (ThermoFisher) and expressed in molar concentrations of molecules if not stated otherwise.

### Fluorescence anisotropy

The fluorescent anisotropy reactions containing 10 nM of DNA probe and the indicated concentration of RecF were carried out, either in the regular RecF buffer (20 mM Tris–Cl pH 7.5, 50 mM NaCl, 10 mM MgCl_2_, 0.1 mM EDTA) (Supporting Figure S1) or in the optimal RecF buffer (30 mM Tris–Cl pH 7.5, 100 mM NaCl, 10 mM MgCl_2_, 0.1 mM EDTA, 1 mM DTT, 30% glycerol, 0.1 mg/ml Bovine Serum Albumin). The second buffer was developed to limit RecF aggregation. Where indicated, ATP and RecR were respectively added at 3 mM and 5 μM. The reactions were incubated at room temperature for 15 min prior to measurement. Fluorescent polarization was measured with a Beacon 2000 Fluorescent Polarization System (PanVera, Invitrogen) in static mode set at 25°C. The anisotropy values (*A*) were calculated from the fluorescent values (*P*) observed using the following Equation (1):


(1) $$\begin{eqnarray*}A\ = \frac{{2\ \times \ P}}{{3 - P}}\ \end{eqnarray*}$$

The anisotropy values (normalized anisotropy) expressed in mA were obtained by subtracting the value observed for probe alone from the values observed for the samples containing in addition the indicated RecF concentration. Since the basal anisotropy (probe alone), slightly increased with the length of the DNA substrates, a smaller Bmax could be expected for longer DNA relative to the shorter DNA. The average values and standard error of at least three replicates were plotted in GraphPad PRISM and the specific binding of each reaction was determined using the Hill Slope fit. As described by Equation (2), the Hill Slope fit allows for the calculation of the maximum specific binding value (*B*_max_), the equilibrium dissociation constant (*K*_d_), and the cooperativity factor (*h*).


(2) $$\begin{eqnarray*}Y\ = \frac{{B_{\rm max}\ \times \ {X^h}}}{{K_{{\rm d}}^h\ + \ {X^h}}}\ \end{eqnarray*}$$

For each binding curve, the different components of the binding predicted by the fit with 95% confidence interval (CI; the highest error of the CI is provided in the table), as well as the goodness of the fit (*r*^2^) are listed in the binding Tables 1 and 2.

Importantly, the total intensity was monitored for each condition. If not further mentioned, no significant change was detected. In conditions where binding was observed to affect the total intensity of the probe, further analysis was carried out to determine binding statistics. We calculated the observed quenching effect using the following Equation (3):


(3) $$\begin{eqnarray*}Qi\ = \frac{{\left( {{I_0} - \ {I_i}} \right)}}{{{I_0}}}\ \end{eqnarray*}$$

where *I*_0_ is the total intensity observed in absence of protein and *I_i_* is the total intensity observed in the presence of the indicated concentration of RecF. If the *Qi* observed is negative, an enhancement is observed. A quenching is detected if the value observed is positive. The quenching curves were plotted as a function of RecF concentration as a secondary measure of binding.

### ATPase activity assay

The RecF ATPase activity was tested in the same reaction buffer used for the fluorescence anisotropy assay, except that BSA was omitted and reactions were carried out at 37°C instead of room temperature in order to better detect the weak RecF ATPase activity. ATP hydrolysis was measured by the coupled reaction assay using a CARY 300 Bio (from Agilent) spectrometer. Briefly, pyruvate kinase (PK) and lactate dehydrogenase (LDH) were used to regenerate ATP from ADP. Phosphoenolpyruvate (PEP) and the oxidation of NADH was followed by a decrease in absorbance at 340 nm. Therefore, in addition to the reaction buffer components, ATPase reactions also contained 3 mM ATP, 3 mM PEP, 10 U/ml PK, 10 U/ml LDH, 2 mM NADH and 100 μM nt of unlabeled DNA. Reactions were preincubated 10 min at 37°C before adding 2.5 μM RecF. When present, RecR was added at 5 μM. The concentration of hydrolyzed ATP was expressed in μM and was plotted as a function of time after protein addition. Curves represent the mean values obtained in triplicate for each condition. The observed hydrolysis rates obtained for each experiment of each condition were also plotted.

## RESULTS

This study investigated RecF binding to DNA substrates by fluorescence anisotropy. Fluorescent anisotropy is a powerful tool widely used to study protein-protein and protein–DNA/RNA interactions (32,64,65). Fluorescence anisotropy allows quantitative measurement of the binding affinity in reactions at equilibrium. When excited by a polarized light, small fluorescent molecules (with an intrinsic fluorescence provided by a tryptophan residue or an additional fluorescent label) tumble rapidly in solution. This leads to a low anisotropy due a greater rotational diffusion of the fluorescent signal captured. Upon interaction with larger molecules, tumbling of the complexed molecule is reduced, and the anisotropy increases. Fluorescence anisotropy increase hyperbolically with the increase in mass of the complex. Thus, anisotropy isotherm titration binding curves allow the determination of the binding affinity and the maximum binding. The method detects cooperativity in binding. It can also help determine the stoichiometry of a complex for a probe with sufficiently small mass relative to the final complex (64–66). Fluorescence anisotropy is particularly suitable to determine, directly and readily, interactions with binding affinities that fall in the range of nM to μM. There are a few limitations. Often, fluorescent labelling of the molecule is required and labels can affect the measured affinity of the interaction (64). Fluorescent anisotropy is better suited to study interactions with simple binding events as the fit is done on the global equilibrium and subspecies cannot be separated. Additionally, change in the total fluorescence due to quenching effects can be observed and this can affect the slope of the isotherm binding curve (65). In such cases, binding affinities can be obtained directly by the intensity change curve. These limitations were considered and incorporated as needed when analyzing the data presented below.

Over 40 different DNA substrates were used, many mimicking intermediates generated either during replication or the repair of post-replication gaps. We designed DNA substrates with lengths varying spanning 20–50 nucleotides or base pairs, and as single, double-stranded, linear and circular. Many incorporated gaps or single-strand extensions (ext) mimicking gap ends. A full list of the substrates used in this study is provided in the Materials and Methods. FAM labelled DNA were incubated with increasing concentrations of *E. coli* RecF to investigate the binding of RecF to each substrate. To determine accurately the energetics of RecF binding, the total intensity was also closely monitored upon binding for each condition tested (65). When significant changes in total fluorescence intensity were detected, these were followed to directly evaluate the binding. Except as noted, all experiments were carried out at 25°C. The contribution of co-factors (ATP or RecR) on the RecF binding affinity to those substrates was also tested. The binding parameters obtained from each binding curve are summarized in Tables 1 and 2. Parameters include the apparent *K*_d_, *B*_max_ (the maximum fluorescence intensity observed in a given experiment that was used in binding calculations), and the Hill coefficient *h* to assess binding cooperativity. As an indirect indicator of RecF and RecFR binding to ssDNA extension, RecF ATPase activity was further tested by using unlabeled DNA (at 37°C rather than 25°C in order to better monitor the weak ATPase activity of RecF). In the following sections, the results in Tables 1 and 2 are broken down to focus on important substrate comparisons.

### RecF binding to DNA is ATP dependent

Previous studies revealed that RecF rapidly aggregates at concentrations near μM levels when incubated in an activity buffer used in prior work (23,63,67) consisting of 20 to 50 mM of buffering solution (EPPS, MES or Tris–Cl), 50 mM salt (NaCl or KCl), with 10 mM MgCl_2_, 0.1 mM EDTA, 0.1–1 mM of reducing agent (dithiothreitol or TCEP) and glycerol (5–10%). This aggregation is irreversible and leads to a total loss of DNA binding. Therefore, DNA binding assays were carried out under standard conditions demonstrated to limit RecF aggregation (23,63). These include a higher concentration in glycerol (30%) and salt (100–150 mM) with the addition of Bovine serum albumin (0.1–2 mg/ml). To ensure consistency with earlier results, DNA binding experiments were carried out side by side with the two buffers (‘classic’: low glycerol/salt and ‘optimal’: higher glycerol/salt) using 20mer duplex DNA and nM RecF concentrations to limit RecF aggregation (Supporting Figure S1). While the difference in viscosity resulted in a decrease in the maximum specific binding (*B*_max_) observed in standard conditions, no change in affinity was detected between the two buffer conditions. In the presence of ATP, RecF forms dimers on dsDNA (32,48,53). Previous work suggests possible cooperativity in dsDNA binding (68). Binding curves obtained in the two buffers were fitted using models consistent with either one binding site or cooperative binding. The cooperative model provided a better fit for both binding curves, yielding *h* values of 1.8 and 2.4 for the buffer used previously as standard and the optimized buffer, respectively.

**Figure 1.**
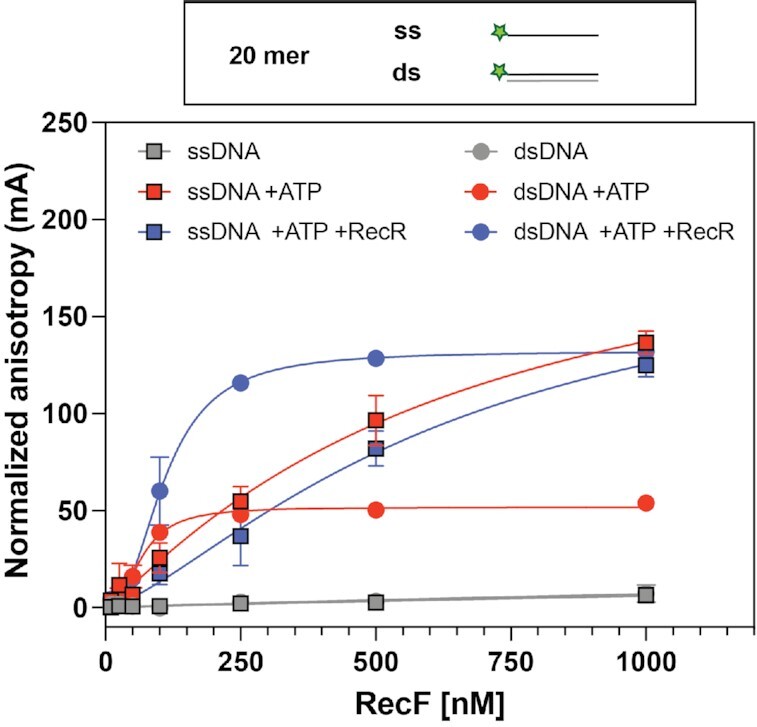
RecF binding to 20mer substrates; ATP dependence. Binding to 20 mer ssDNA (square) and dsDNA (circle) harboring a 5′ FAM label was tested by fluorescence anisotropy. RecF binding was tested alone (grey), or in the presence of 3 mM ATP (red), or in the presence of 3 mM ATP and 5 μM RecR (blue). The values plotted represent the mean of reactions carried out in triplicate and the standard deviation obtained for each RecF concentration. The curves indicate the Hill slope binding model fit obtained for each condition.

After this validation of experimental conditions and determination of the best fitting model, we carried out a straightforward comparison between 20mer dsDNA and a ssDNA oligonucleotide of the same length (labelled on the 5′ end), to determine the binding preference of RecF and the effects of cofactors previously shown to influence its binding (Figure 1). When ATP was omitted from the reaction, no binding was observed for either substrate. On the other hand, binding was detected with both ss and dsDNA when ATP was added. This recapitulates the binding characteristics observed for the *D. radiodurans* and *T. tengcongensis* RecF proteins using DNA substrates of similar length (32,53). A stronger binding to dsDNA was observed with a *K*_d_ of ∼67 nM compared to ∼640 nM for ssDNA, but a lower *B*_max_ 51.8 mA versus 220.7 mA (estimated) for ssDNA. The *h* values observed of 2.4 for dsDNA and 1.1 for ssDNA agreed with the multimeric state expected on ds and ssDNA. Finally, on dsDNA the addition of RecR substantially increased the B_max_ to 132.1 mA and slightly reduced the measured affinity (*K*_d_ = 109 nM), while having no effect on the ssDNA binding. The increase in *B*_max_ observed upon RecR addition suggest a larger complex and could reflect either a protein conformational change or a change in the size of the RecF binding site.

Together these results suggest an ATP dependent binding of RecF to DNA and further confirmed the preferential binding to 20mer dsDNA in the presence of ATP that was observed previously (32,53,63). The observed cooperativity value observed is compatible with RecF dimerization. The increase in specific binding observed when RecR is added is consistent with RecR-mediated stabilization of RecF binding to dsDNA. This increase in specific binding highlights the differential effect RecR has on RecF dsDNA binding (Figure 1).

### RecF binding to ssDNA is affected by DNA length

**Figure 2.**
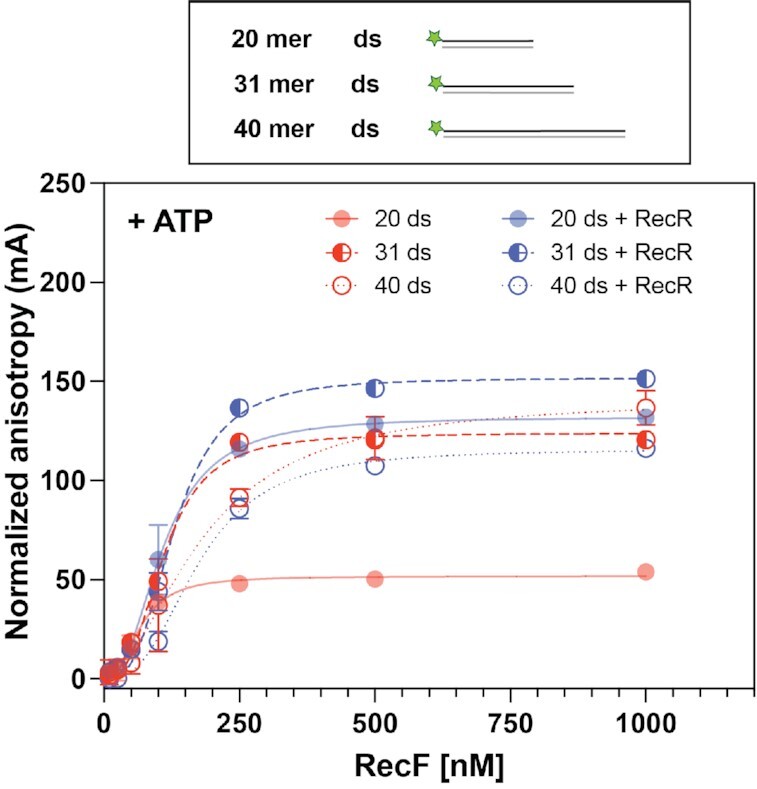
RecF binding to dsDNA; length effect. RecF binding to 20 (filled circle, full line), 31 (half-moon, dashed line) and 40 mer (empty circle, dotted line) dsDNA FAM labeled on one 5′ end was tested by fluorescence anisotropy. Binding was tested in the presence of 3 mM ATP (red), or in presence of 3 mM ATP combined to 5 μM of RecR (blue). The values represent the mean of reactions carried out in triplicate and the standard deviation obtained for each RecF concentration. The curves represent the Hill slope binding model obtained for each condition. For the 20mer, the values used in the graph were the same as presented in Figure 1. They are provided for ease of comparison and are depicted with 50% transparency.

We next examined the effect of DNA length on RecF binding. Subtle effects of DNA length on RecF and RecFR binding have previously been noted for the RecF and RecR proteins from *D. radiodurans* (32). In order to determine whether the length of the DNA also affects *E. coli* RecFR DNA binding, RecF and the effects of RecR were examined using longer dsDNA substrates (31- and 40-mers, Figure 2). In the absence of RecR, a slight decrease In RecF binding affinity was observed for longer dsDNA substrates. A *K*_d_ of 108.2 and 174.6 nM was observed for the 31- and 40-mer, respectively. Additionally, an increase in the cooperativity index to about 3, as well as an increase in the specific maximum binding signals (to 162.4 and 181.9 mA) was observed. This might be expected for longer DNAs, which potentially allow binding of a larger number of RecF. The addition of RecR appeared to have less effect as the dsDNA became longer. On the 31mer, RecF stabilization mediated by RecR is still detected but is smaller compared to the effect observed on the 20mer. With the longer 40mer DNA, no change in *B*_max_ was detected; however, an increase in cooperativity was observed in the presence of RecR.

**Figure 3.**
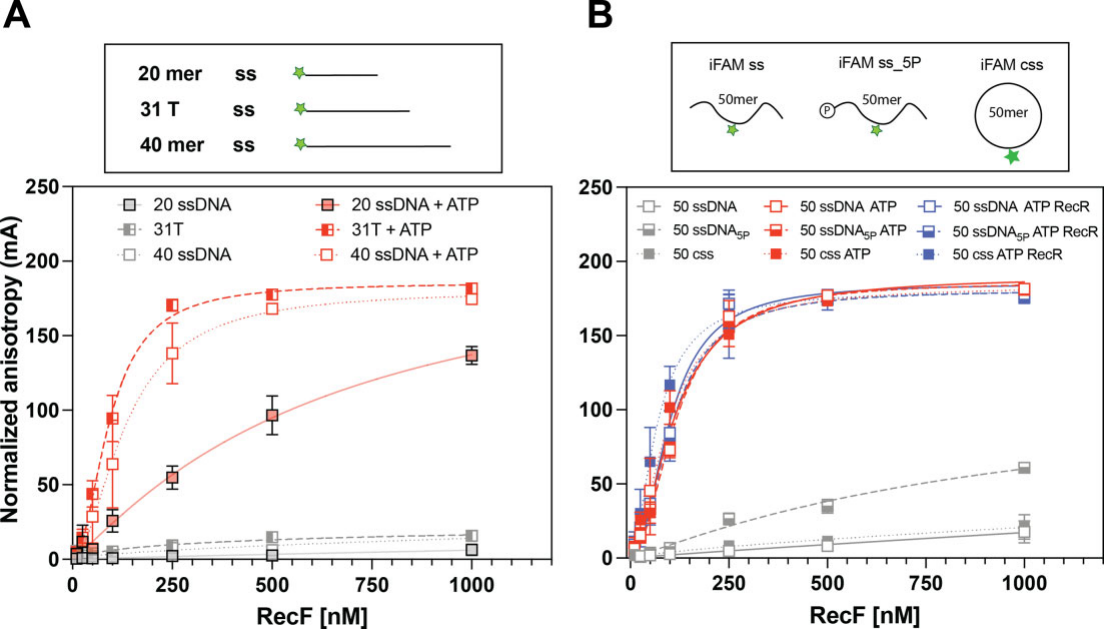
RecF binding to ssDNA(s); length effect. The RecF binding to ssDNA varying in length was tested by fluorescence anisotropy. The values represent the mean of reactions carried out in triplicate and the standard deviation obtained for each RecF concentration. The curves represent the Hill slope binding model obtained for each condition. (**A**) RecF binding to 20 (filled square, solid line), 31 (half-filled square, dashed line) and 40mer (empty square, dotted line) ssDNA FAM labeled on the 5′ end was tested was tested either in the absence (grey) or in presence of 3 mM ATP (red). For the 20mer, the values used were the same in Figure 1 and are presented with 50% transparency. (**B**) RecF binding to 50mer ssDNA substrates non phosphorylated (open square, solid line), 5′ phosphorylated (half-filled square, dashed line) and circularized (filled square, dotted line) was tested in absence of co-factors (grey), in presence of 3 mM ATP (red) or in presence of 3 mM ATP and 5 μM of RecR (black).

Notably, the binding of RecF to ssDNA increased substantially when the oligonucleotide substrates were lengthened. Binding was examined in the presence of ATP on a 31polydT substrate and a random sequence 40mer, both FAM labeled on the 5′ end. The RecF binding curves (Figure 3A) revealed a dissociation constant of 93 and 134 nM, respectively, for these two ssDNAs. The length increase thus increases binding affinity by about 6-fold.

Reports about RecF binding to ssDNA feature a number of disparities (23,32,51,53,69). RecF proteins purified from different organisms including *E. coli* were found to bind ssDNA in an ATP dependent fashion, using gel shift, fluorescence anisotropy or interferometry layer assays, as observed in Figure 1 (23,32,53). However, earlier studies on *E. coli* RecF binding using electrophoresis mobility shift assay and filter binding assays observed a measurable ATP independent binding (51,69). We noted that the main difference in those assays was the radiolabeling on the 5′ end with P^32^ in the earlier studies, where only oligonucleotides 5′ FAM labeled and/or lacking 5′ phosphorylation were used in the more recent studies. As RecF binds ATP and phosphocellulose resin (51,53,70), we hypothesized that RecF binds single stranded DNA in the absence of ATP if the DNA is 5′ phosphorylated. Therefore, we designed a new 50-mer oligonucleotide, called iFAM ss_5P, with 5′ phosphorylation and in which FAM was labeled internally at position 25 (iFAM) (for full sequence see Materials and Methods). This 50mer iFAM ss_5P was used to obtain two more variations—a dephosphorylated version (iFAM ss) and a circular 50mer (iFAM css). Proper circularization was verified by lack of degradation when treated with ExoI (Supporting Figure S2A). In the absence of ATP, weak RecF binding was detected with the 5′ phosphorylated ssDNA (Figure 3B). The dissociation constant was estimated to be nearly in the μM range and the cooperativity factor was determined to be 1. Binding with the dephosphorylated and circularized versions of this oligo was essentially undetectable. The addition of ATP was found to substantially increase RecF binding to all three ssDNA substrates with observed *K*_d_ values of 115.7, 112.4 and 97.8 nM for iFAM ss_5P, iFAM and iFAM css, respectively. In presence of ATP the small effect of the 5′ end phosphorylation increasing binding disappeared. For all, an increase in the cooperativity index to reach a value near 2 was observed. Finally, the addition of RecR was found to have very little effect, slightly decreasing the *K*_d_ to 68.4 nM for iFAM css. Importantly, the ATP independent binding described above for 5′phosphorylated ssDNA, was not observed with double stranded DNA (Supporting Figure S2B). Altogether, these results revealed that (i) RecF binds weakly to 5′ phosphorylated ssDNA in the absence of ATP, (ii) binding to DNA was strongly ATP dependent for all substrates, (iii) disparities in binding to dsDNA and ssDNA largely disappear when the DNAs are lengthened beyond 20 bp or nucleotides and (iv) RecF binding to dsDNA is somewhat affected by RecR but binding to ssDNA is largely RecR independent. The effects of length on ssDNA binding suggest a binding site size minimum between 20 and 31 nucleotides. Except for the shortest ssDNAs, measured *K*_d_ values fell in a range between 67 and 198 nM for RecF binding to these substrates.

### Effects of DNA end labels

**Figure 4.**
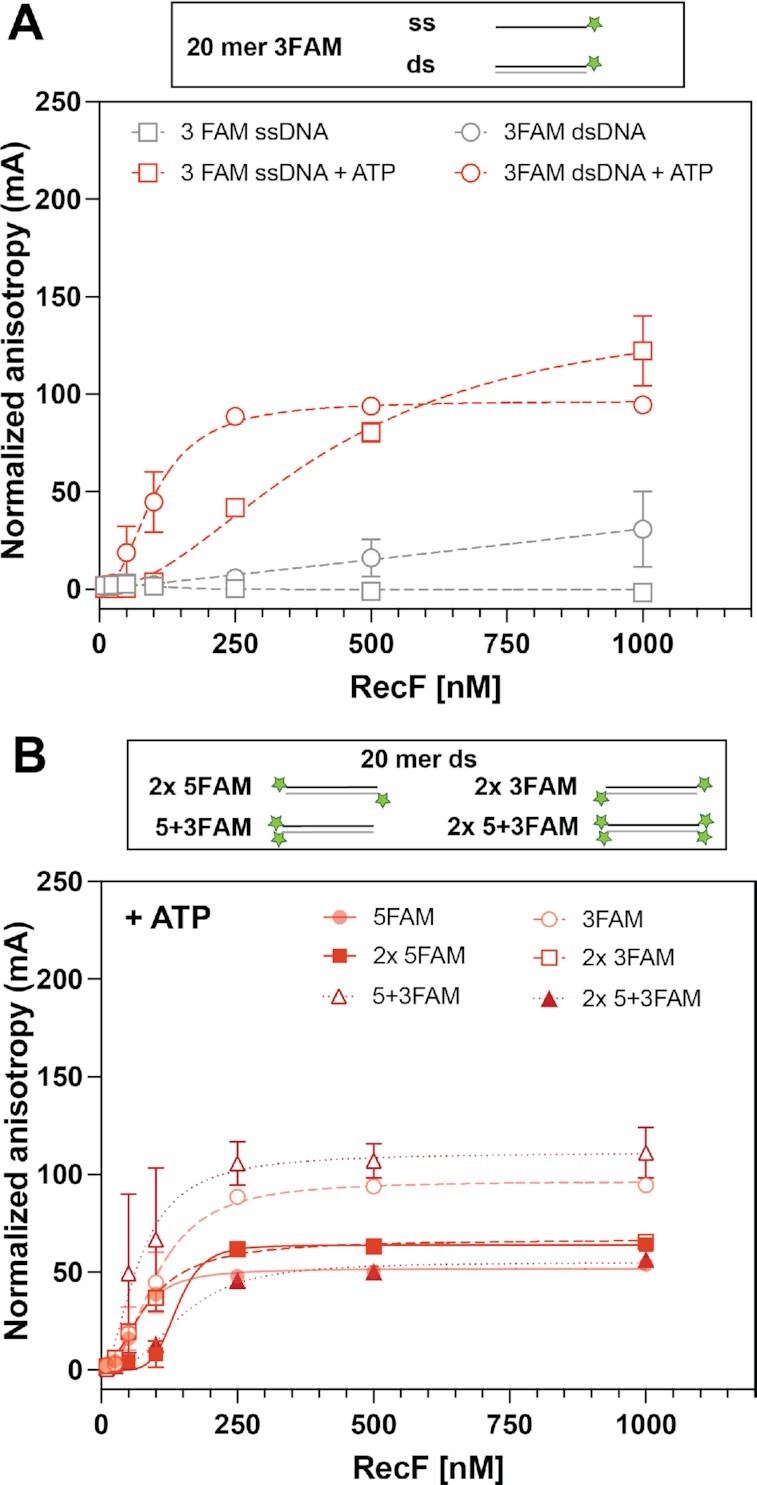
Effect of DNA end FAM labels. RecF bnding to 20mer DNA substrates presenting variation in their FAM labelling orientation was tested by fluorescence anisotropy. The values plotted represent the mean of reactions carried out in triplicate and the standard deviation obtained for each RecF concentration. The curves represent the Hill slope binding model obtained for each condition. (**A**) RecF binding to 3FAM ssDNA (empty square), or dsDNA (empty circle) was tested in absence (grey), or in presence of 3 mM ATP (red). (**B**) RecF binding to dsDNA substrate presenting several ends FAM labelled, i.e. 2 × 5FAM (filled square; solid line), 2 × 3FAM (open square, dashed line), two FAM labels on the same DNA end called 5 + 3FAM (open triangle, dotted line) and 2 FAM labels on both ends (filled triangles; dotted line) were carried out in the presence of 3 mM ATP. Binding curves of multiple labeled DNA are compared to the curves previously obtained for the singly labeled versions (from Figures 1 and 4A) presented with 50% transparency.

The FAM label used to monitor DNA binding is a large molecule that could itself affect binding if there is a binding specificity involving DNA ends, or if a free end simply facilitates RecF access to DNA via sliding. We thus varied the positioning of the FAM label to determine if the binding results were affected. The trials included 20mer dsDNA substrates labeled with FAM on one 5′ or one 3′ end. Multiple FAM labels were also used for some DNAs, with 2 or 4 labels added in every possible combination. The results are presented in Figure 4 and Table 1. The FAM labels produce some subtle effects on DNA binding, significantly when both 5′ ends are labeled on the dsDNA substrate. Most of the variation is within the range seen when DNA lengths or other features of the DNA structure are changed (67–147 nM in this set of experiments). The one exceptional result is a significant increase to ∼5.8 in measured cooperativity when both 5′ ends are labeled. This may reflect an effect of DNA ends for RecF access to DNA, although ends are not required for binding.

For both substrates 5 + 3FAM and 2 × 5 + 3FAM (note 5 + 3 designates substrates with FAM labels on both strands of the same dsDNA end), the change in fluorescence anisotropy was accompanied by a change in maximum intensity (Imax). This suggests that RecF is binding near the FAM label in these substrates, whereas the FAM label was avoided in substrates labeled on only one end. This change in intensity was in part a quenching effect and is plotted as quenching intensity observed over the increasing RecF concentration (Supporting Figure S3). Dissociation constant values obtained from the quenching intensity were not significatively different from the anisotropy curve. The only difference observed was an increase in the *h* value from ∼3 to 4 for the 2 × 5 + 3FAM substrate.

### The effect of ssDNA extensions

**Figure 5.**
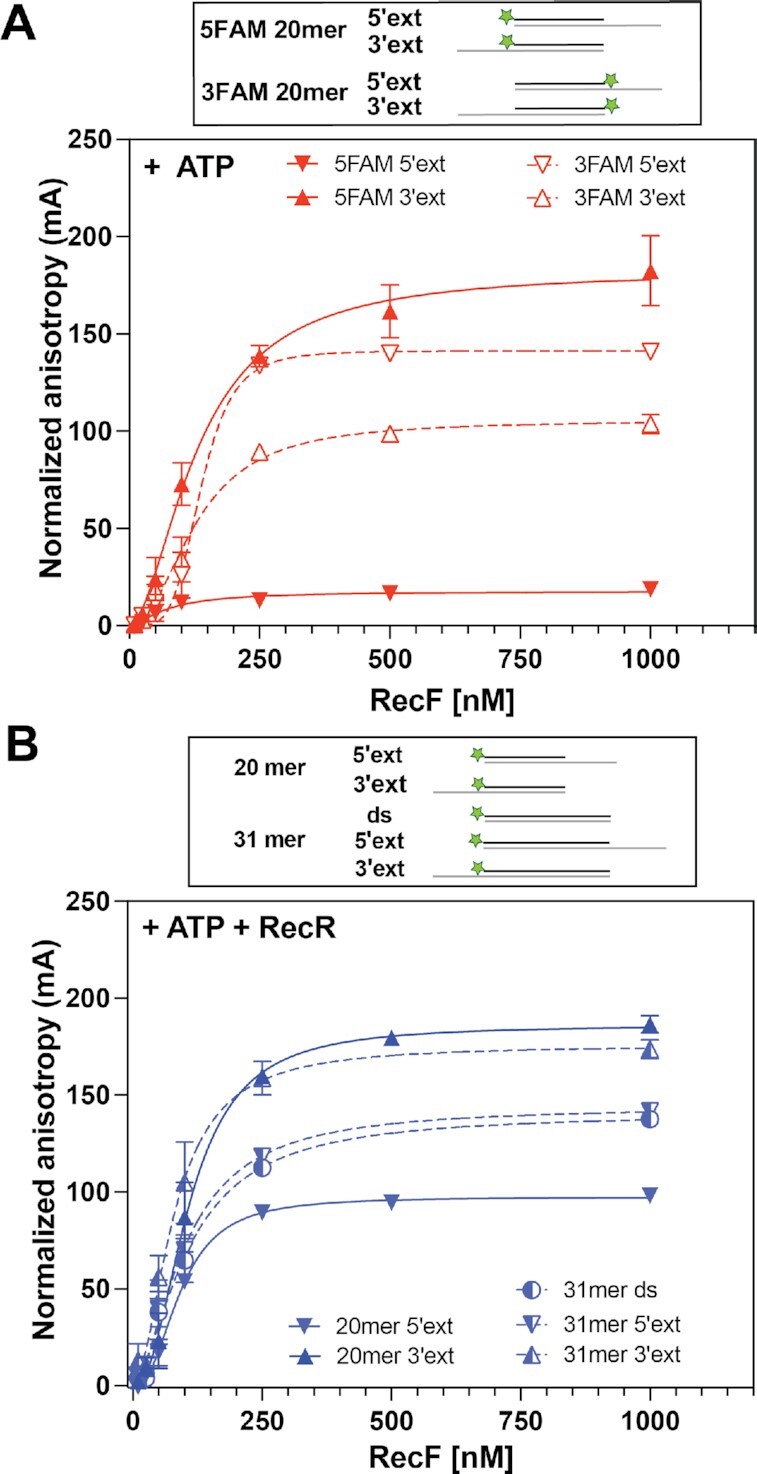
Effect of ssDNA extensions on RecF binding. Binding to dsDNA substrates with a ssDNA extension composed of 12T on the 5′ or 3′ end, as well as variation in their FAM labelling orientation, was tested by fluorescence anisotropy in the presence of (**A**) 3 mM ATP or (**B**) 3 mM ATP and 5 μM RecR. The values represent the mean of reactions carried out in triplicate and the standard deviation obtained for each RecF concentration. The curves represent the Hill slope binding model obtained for each condition. (**A**) RecF binding to 20mer ds with a 5′ extension is represented as down triangle (solid for 5FAM and open for 3FAM) while binding to 20mer dsDNA with a 3′ extension is represented as top triangle (solid for 5FAM and open for 3FAM). (**B**) RecF binding to a 20mer (filled triangles; solid lines) and 31mer dsDNA (half-filled circle; dashed lines) without or with either a 5′ or 3 'extension as indicated in the schematic.

We next explored RecF and RecFR binding to a wide range of duplex DNAs with either 5′ or 3′ single stranded extensions (ext) designed to mimic gap ends (Table 1 and Figure 5). FAM labels were positioned in a variety of locations in the various substrates and ssDNA extension lengths were varied from 3 to 12 nucleotides. In general, the binding *K*_d_ observed for RecF or RecFR varied over a 2.5-fold range from 76 to 195 nM, a range again very similar to that seen with the other DNA substrates described above. The one structural change that produced the largest change in binding was the inclusion of a 5′-phosphate group at the exposed 5′ ends of two substrates (Table 1). This caused a significant increase in *K*_d_ or decline in affinity. The strongest binding was seen for one substrate with a 5′ extension and a free 3′ hydroxyl (-OH) on the gap end, although this remained well within the range seen with substrates with other structures. *B*_max_ also strongly declined for this substrate (Figure 5A). The length of the 3′ extension was found to reduce the *B*_max_ by about 40% for the extensions of 3 and 6 nucleotides (Supporting Figure S4). The reduction in *B*_max_ (Figure 5A) was strongly relieved by the addition of RecR (Supporting data Figure S5). This result suggests a conformation change or other stabilization effect brought on by the interaction with RecR. The addition of RecR to these experiments had modest, if any, effects on other measured binding parameters (Table 1 and Figure 5B), particularly the measured *K*_d_.

**Figure 6.**
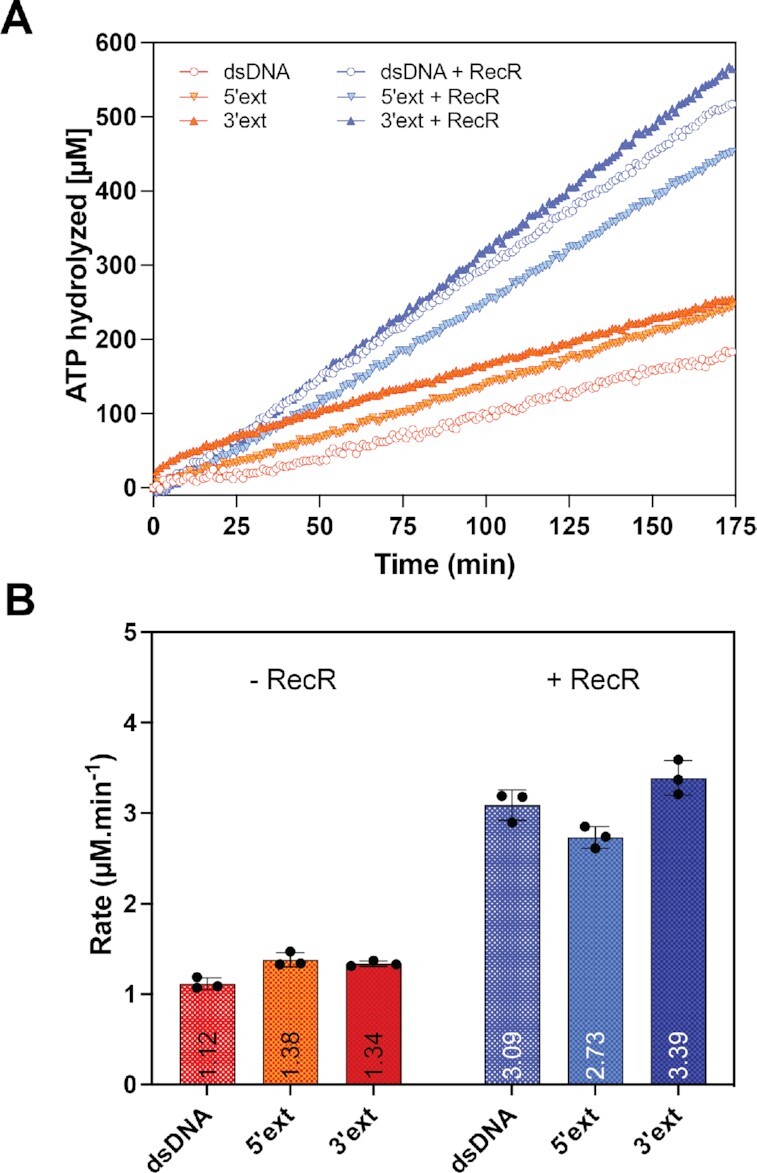
Effect of ssDNA extensions on the RecF ATPase. The ATPase activity of RecF (2.5 μM) was tested in presence of 31mer dsDNA with or without a 5′ or 3′ extension (ext). When indicated, 5 μM of RecR was added to the reactions. The top panel (**A**) represents the average traces of reactions carried out in triplicate for the ATP hydrolyzed as function of time and the bottom panel (**B**) provides average rates obtained for each condition with individual values of the triplicate appearing as dots.

To complement this data and look further for effects of RecR on RecF, the ATPase activity of RecF was tested upon binding to the unlabeled version of the 31dsDNA with or without 5′ or 3′ extensions (Figure 6). ATPase assays were carried out in the same reaction buffer but the temperature was increased to 37°C, due to the already weak ATPase activity. In the absence of RecR, ATP hydrolysis rates were respectively 1.12, 1.38 and 1.34 μM min^−1^ for dsDNA, and dsDNA with 5′ or 3′ extensions, respectively. Thus, other than the large increase in *B*_max_ we observed in the DNA binding experiments when RecR was added for one substrate, we detected little effect of RecR on RecF binding to dsDNA substrates with single strand extensions. We also detected no substantial effect of the ssDNA extensions on measured rates of ATP hydrolysis.

### Effects of a cyclobutane pyrimidine dimer

**Figure 7.**
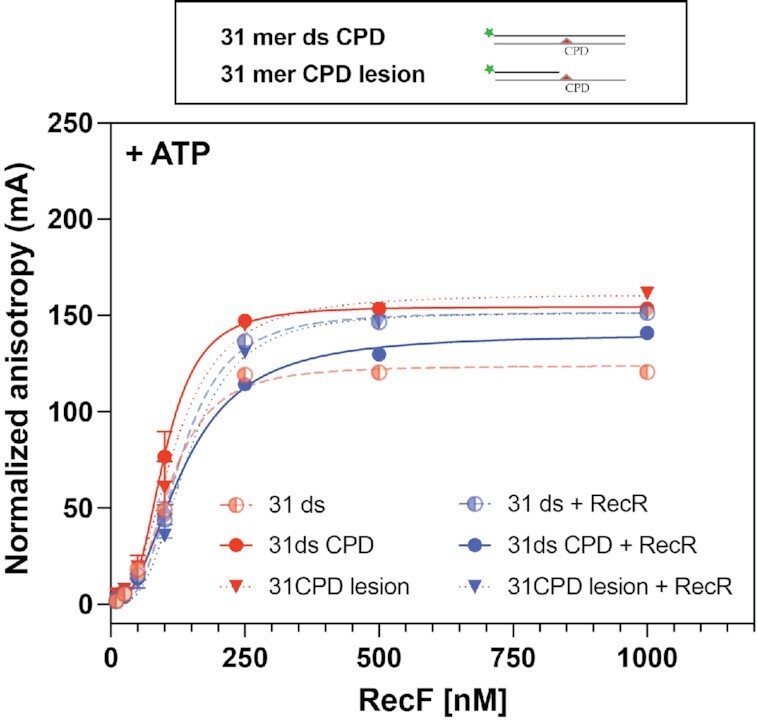
RecF and RecFR do not exhibit stronger affinity for DNA substrates containing a CPD lesion. The RecF binding to DNA substrates presenting thymine dimer was tested in presence of 3 mM of ATP alone (red) or in combination with 5 μM RecR protein (blue). The binding was tested for a 31mer dsDNA in which a thymine dimer is in the middle of the sequence (filled circles, solid lines), called 31mer ds CPD and for its variation in which the thymine dimers is present in the 15 nucleotides 5′ extension ssDNA flanking a 16 bp dsDNA region (filled triangles, dotted lines), called 31mer CPD lesion. The binding curves to CPD substrates were compared to the value previously obtained for the 31mer dsDNA (Figure 2), represented here with 50% transparency (half-filled circle, dashed lines).

A lesion, such as a cyclobutane pyrimidine dimer (CPD), should be present in a post-replication gap, possibly immediately adjacent to the 3′ gap end where replication was halted and the polymerase disengaged. In principle, if RecF is not binding specifically to gap ends, targeting of RecFOR to gaps could be brought about by specific binding of lesions at such gap ends. The i*n vitro* assays used to test RecF DNA binding activities have all previously used DNA substrates lacking a lesion of any kind (23,24,31,32,53). We sought to determine whether RecF binds preferentially to DNA harboring a cyclobutane pyrimidine dimer. The lesion was positioned either at the middle of a 31 mer dsDNA, or to a shorter 5FAM labeled DNA consisting of a 16 nucleotide duplex with a 15 nucleotide 5′ single strand extension with the lesion positioned immediately adjacent to the 3′ end of the shorter strand. The latter was designed to mimic the presumed structure formed when the replication machinery encounters a CPD lesion. The binding of RecF to these substrates was tested in the presence of ATP, with or without RecR protein (Figure 7 and Table 2). The resulting binding curves closely resembled those obtained with other DNA substrates. The RecR protein increased the *K*_d_ measured for both substrates to a small extent. However, all binding parameters remained well within the ranges established for substrates examined in Table 1. These results provided no evidence for preferential binding to a CPD DNA lesion.

### Effects of more complex DNA structural alterations

**Figure 8.**
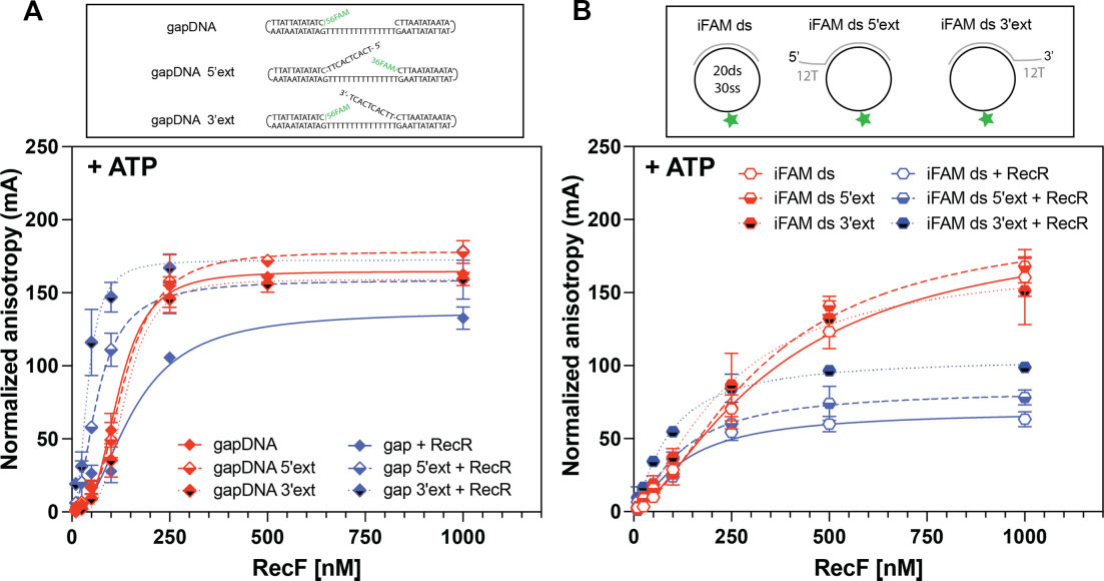
RecF binding to gapped DNA with or without unpaired ssDNA extensions. The RecF binding to DNA substrates presenting a single strand gap with or without an additional 5′ or 3′ extension facing the gap (but not complementary to it), was tested by fluorescence anisotropy in presence of 3mM ATP alone (red) or in combination with 5μM of RecR (blue). The values plotted represent the mean of a triplicate and the standard deviation obtained for each RecF concentration. The curves represent the Hill slope binding model obtained for each condition. (**A**) The graph represents the binding curves obtained for the RecF binding to a single strand gap formed in between two hairpins and its two modified versions with either with a 5′ or 3′ unpaired ssDNA extension. (**B**) Binding curves obtained for the RecF binding to circular single strand gaps of 30T internally FAM labeled with a 20 bp dsDNA region and its two modified versions with either with a 5′ or 3′ unpaired ssDNA extension.

We also constructed a series of DNA substrates containing short (15 or 30 nucleotide gaps flanked by duplex at both ends, by using hairpins at each end or using a circularized ssDNA (Figure 8 and Table 2). Two of the hairpin substrates each had either a 5′ or 3′ unpaired single strand extension similar to substrates used in an earlier study (15). For the hairpin substrates and in the absence of RecR, the measured binding patterns generally fell into the ranges established in Table 1. However, when the hairpin substrates had single strand unpaired extensions, RecR addition improved binding significantly, on the order of 3-fold (Figure 8A and Table 2). This may reflect a previously undetected binding preference by RecFR. The binding parameters remained close to the stronger binding patterns seen in Table 1. A mild but significant intensity quenching was observed for the substrate containing the 5′ single strand extension, suggesting that RecR-mediated binding occurred close to the 3FAM labeled end of this substrate (Supporting Figure S6). The binding parameters determined with the change in intensity were similar to the parameters obtained with the anisotropy curve.

On its own, RecF bound poorly to the circularized DNA substrates (Table 2 and Figure 8B). The reduction in binding might reflect the positioning of the FAM label in the middle of the ssDNA gap or it might reflect the absence of a DNA end. There is a change in intensity observed upon binding, that suggests RecF binds to the ssDNA region carrying the FAM (Supporting data Figure S6). Here again, the binding parameters deduced from the change in intensity were similar to the value obtained from the anisotropy curve. Addition of RecR brought the binding patterns well within the ranges established in Table 1. FAM signal quenching was also reduced in the presence of RecR suggesting a re-localization of RecFR away from the iFAM, i.e. on the dsDNA region (Supporting Figure S6). Effects of RecR are prominent with these substrates, indicating that RecR facilitates RecF binding to this small circularized substrate.

### Effects of nicks and short gaps

Perhaps the most common gap structures to be found in genomic DNA are the nicks and very short gaps associated with nucleotide excision repair and base excision repair. We thus tested the binding of RecF and RecFR to duplex DNA substrates, 31 bp long, containing either a nick or 31mer composed of 30 bp presenting one nucleotide gap in the middle of one strand. As presented at the end of Table 2 and Supporting Figure S7, the binding parameters for both RecF and RecFR were well within the range observed for binding to the other substrates used in this study.

## DISCUSSION

In some manner, the RecFOR system is targeted to and directs recombinational DNA repair within lesion-containing post-replication gaps. To explain this targeting, the major mechanism proposed to date is a specific binding of RecF and/or RecFR to the ends of gaps. As this targeting hypothesis does not incorporate a mechanism to distinguish between lesion-containing gaps that require repair and the many other gaps associated with DNA metabolism that do not require repair, we have systematically explored the DNA binding properties of RecF and RecFR. The major conclusion of this work is that there is no evidence for specific binding to the ends of a DNA gap, for either RecF or RecFR, with anything approaching the level of specificity that would be required to target the RecFOR system to rare lesion-containing post-replication gaps. To successfully locate their target sequences in a bacterial genome, DNA binding proteins such as the lac repressor exhibit a binding preference for their specific sequence by factors of 6–7 orders of magnitude relative to random sequence DNA (71,72). Targeting the RecFOR system to rare post-replication gaps is no less of a physical problem. In the current study, subtle effects of DNA structure are evident in the measured DNA binding parameters. However, virtually all of the parameters vary over a quite limited range.

This systematic examination of RecF and RecFR binding to over 40 different DNA structures provides several additional observations regarding RecF binding to DNA, many of them subtle. These include (i) a level of cooperativity in RecF and RecFR binding to DNA that may reflect the formation of larger oligomers than previously considered for these proteins, (ii) a strong dependence on ATP, (iii) a stronger affinity for ssDNA than has been evident in some studies, reflecting a required binding site size that is >20 nucleotides, (iv) some subtle effects of exposed 5′-phosphoryl groups, (v) the absence of specific binding to DNA substrates featuring a cyclobutane pyrimidine dimer and (vi) some effects suggesting that RecF binding to DNA may be facilitated to a modest extent by the presence of a free DNA end.

The RecFOR system must somehow find rare lesion-containing gaps. The lack of specific binding to gap ends solves one conundrum. As indicated in the Introduction, specific binding to gap ends could be deleterious. The reason is simple. Lesion-containing post-replication gaps are rare and embedded in a genomic environment where they are vastly outnumbered by the normal lagging strand gaps created during replication. Strongly specific binding of RecF(R) to gap ends not only does not exist if such specificity did exist, it would likely be deleterious. To load RecA into all gaps routinely could trigger many potentially detrimental DNA pairing events behind the replication fork.

If the most obvious unique structural feature of gaps—their ends—does not provide the needed targeting specificity, the RecFOR system targeting mechanism must be found elsewhere. The plethora of *in vivo recF* deletion studies strongly indicate that RecF remains a likely key to targeting. RecF is not itself required for RecA protein loading but decades of research clearly implicates RecF as having an important role in the process. If not DNA, we suggest that the targeting mechanism could involve a specific interaction with a protein that would be found at or near the lesion-containing gap. Three obvious possibilities come to mind. The first is DNA polymerase itself, which acts as the primary sensor in detecting a template lesion and creating the gap via lesion-skipping. The second is SSB, which is expected to coat any ssDNA present in the genome. The third is the RecJ nuclease, which has been implicated in enlarging gaps prior to RecA protein loading (7,8). Unlike RecO, RecF does not interact with SSB (40,69,73,74). An interaction between RecJ and RecF has not been carefully explored, but a RecJ-RecF interaction was not detected in a global pull down screen designed to explore *E. coli* protein interactions globally (75). However, RecF frequently co-localizes with the replisome (36) and that same global pull down screen did detect a possible interaction between RecF and DnaN (albeit amid a host of other potential DnaN and RecF interactions) (36,75). An interaction between RecF and the replisome has been a topic of speculation going back to the 1990s (44,76,77). We explore this potential interaction, and the implications it has for a targeting mechanism, in the accompanying paper (74).

## DATA AVAILABILITY

The data underlying this article are available in the article and in its online supplementary material.

## Supplementary Material

gkad311_Supplemental_File

## Notes

Present address: Neema Mbele, School of Medicine and Public Health, University of Wisconsin-Madison, Madison, WI 53726, USA.
