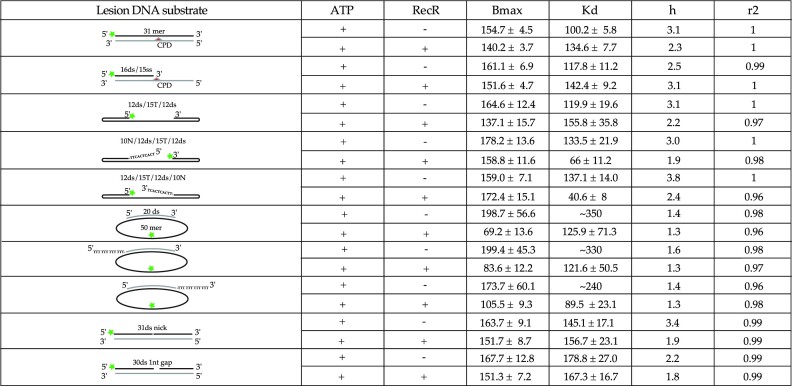# Correction to ‘RecF protein targeting to postreplication (daughter strand) gaps I: DNA binding by RecF and RecFR’

**DOI:** 10.1093/nar/gkad475

**Published:** 2023-05-22

*Nucleic Acids Research*, 2023; gkad311, https://doi.org/10.1093/nar/gkad311

In the originally published version of this manuscript, Table 1 appeared twice, as Table 1 and Table 2. Table 2 was missing.

This error has now been corrected.

**Table 2.** Binding characteristics of Hill-slope fits obtained for DNAs containing CPD or single strand nick or gap region. Indicated in the table: the type of DNA, the schematic of the DNA substrates for which the FAM label is represented by a green star, the CPD lesion by a red triangle and the complementary DNA is indicated in light grey, the presence or not of ATP and RecR, the Bmax values with the higher value of the 95% confidence interval, the *K*_d_ with the higher value of the 95% confidence interval, the cooperativity value (*h*) and the goodness of the fit (*r*^2^)