# Supplementary material for: RecF protein targeting to postreplication (daughter strand) gaps I: DNA binding by RecF and RecFR
Source: Nucleic Acids Res. 2023 May 1;51(11):5699–713. doi: 10.1093/nar/gkad311 (PMC10287957; doi:10.1093/nar/gkad311)
Supplement: gkad311_Supplemental_File [file gkad311_supplemental_file.pdf]

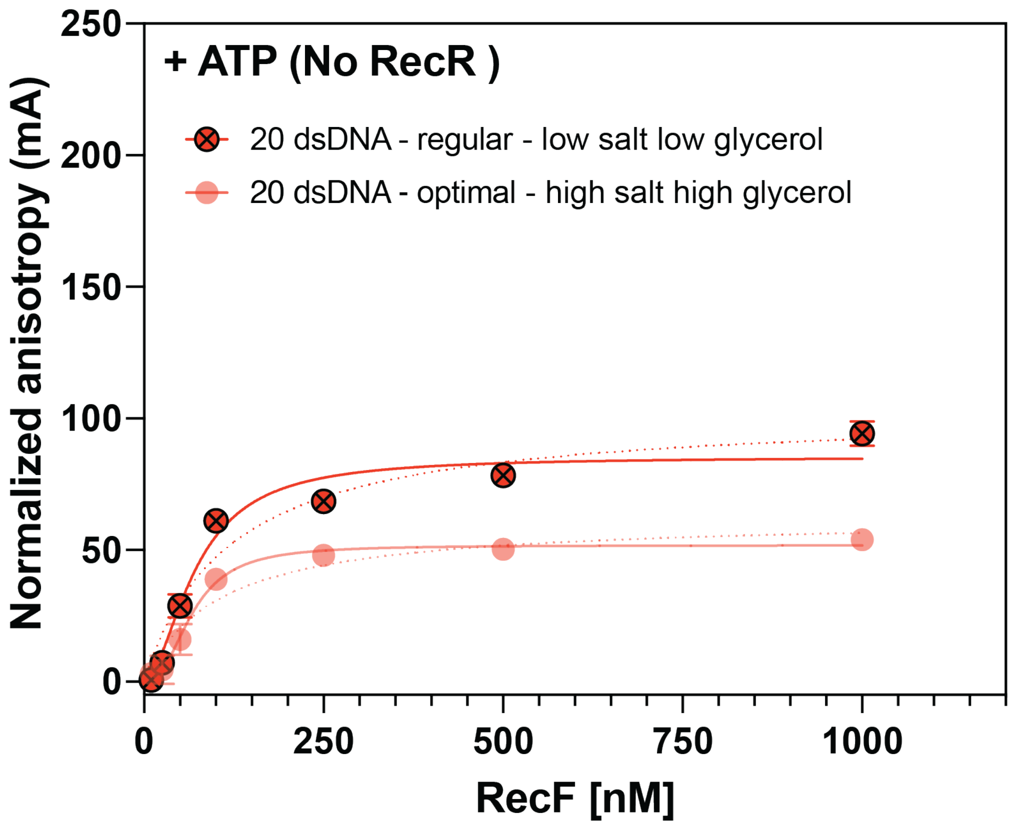

Buffer	Bmax	Kd	h value	r^2	Binding model
Regular	103.1 ± 12.3	117.9 ± 50.7	N.A.	0.94	One binding site
	**85.4 ± 11.6**	71.7 ± 29.6	**1.8**	**0.96**	Hill slope
Optimal	62.3 ± 7.9	102.3 ± 47.7	N.A.	0.92	One binding site
	**51.8 ± 3.2**	67.0 ± 10.0	**2.4**	**0.97**	Hill slope

Supporting figure 1: RecF binding to dsDNA in regular and optimal buffer

The effect of the buffer composition on RecF DNA binding was tested by fluorescence anisotropy in presence of 3 mM ATP and 20mer dsDNA 5FAM. RecF binding was tested in the published buffer (crossed circle), low glycerol and low salt, was compared to the RecF binding obtained in the optimized buffer designed to prevent aggregation, containing a concentration 6x higher in glycerol and twice as high in salt

(same values as presented in Figure1, open circle, 50% transparency). Binding curves represent experiments done in triplicate in both conditions are plotted on the top panel. GraphPad PRISM was used to fit the binding curves either using a One binding site model (dotted line) or the Hill-slope model (full line). Characteristics of the binding fit generated either using the One binding site or the Hill-slope models are reported in the bottom panel table.

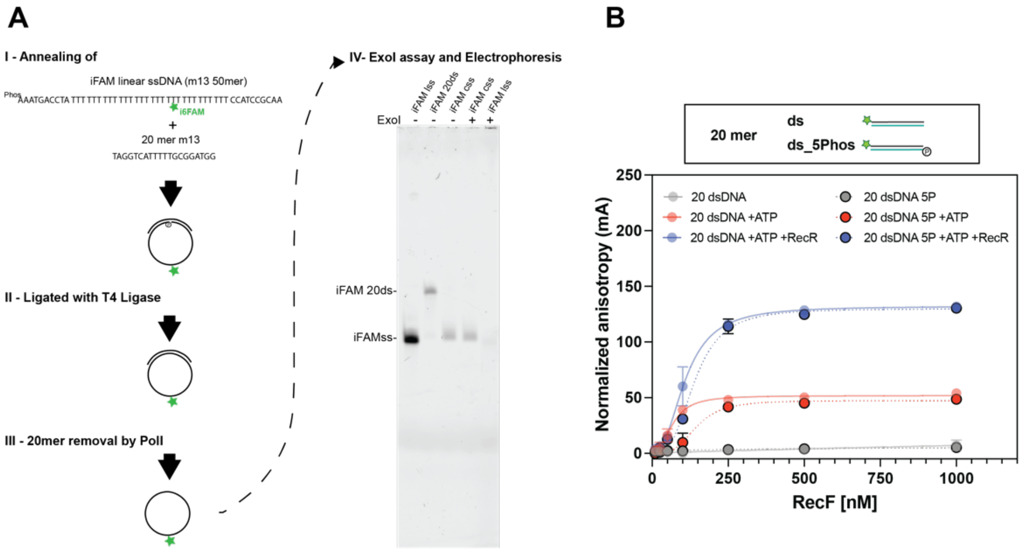

Supporting figure 2: Steps to generate the 50mer circular ssDNA and RecF binding to 5'-phosphorylated dsDNA

A Schematic of the key steps used to generate the circular ssDNA internally FAM labeled, and final electrophoresis gel (12% Native TBE) used to verify the circularization by resistance to ExoI degradation. The "+" and "–" indicate that the samples were submitted or not to ExoI degradation prior to loading. The linar iFAM (lss) was loaded in the first line, followed by the ligated iFAM annealed to 20ds (iFAM 20ds), then the iFAM 20ds treated by PolI which generate the iFAM css, and in the last two lines the css and lss treated by ExoI. **B** The binding of RecF to 20mer 5'phosphoprylated dsDNA (black circle, dot line) was compared to the binding obtained for the 20mer dsDNA non phosphorylated presented in the Figure 1 (plain circle, full line 50% transparency). Binding was tested in the absence of co-factors (grey), in presence of 3 mM ATP (red) or

in presence of 3 mM ATP and 5 μM RecR (blue). The values plotted represent the mean of reactions carried out in triplicate and the standard deviation obtained for each RecF concentration. The curves represent the Hill slope binding model fit obtained for each condition.

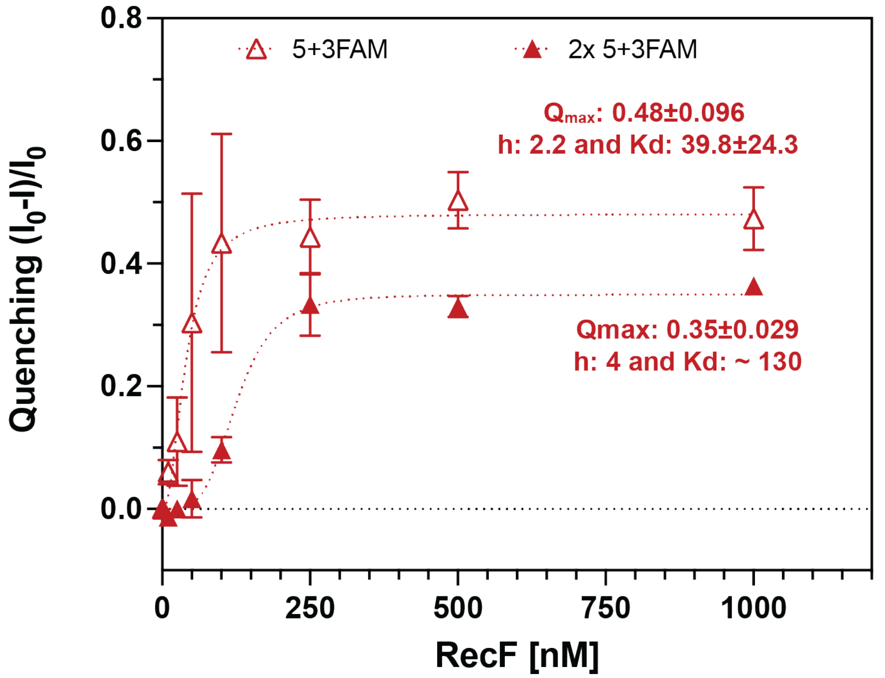

Supporting figure 3: Quenching intensity observed for 20mer dsDNA with two FAM labels on one end.

Quenching intensity observed upon binding to 20mer dsDNA probes, for which 5' and 3'FAM labels are on the same end. RecF binding was tested in the presence of 3 mM ATP. Quenching intensity of 5+3FAM (open triangle) and 2x5+3FAM (solid triangle) were plotted as a function of RecF concentration. The values plotted represent the mean of reactions carried out in triplicate and the standard deviation obtained for each RecF concentration. The curves represent the Hill slope fit binding model obtained for each condition. The parameters obtained from the Hill slope fit include maximum quenching (Qmax), the cooperativity index (h) and the estimated dissociation constant (K_d).

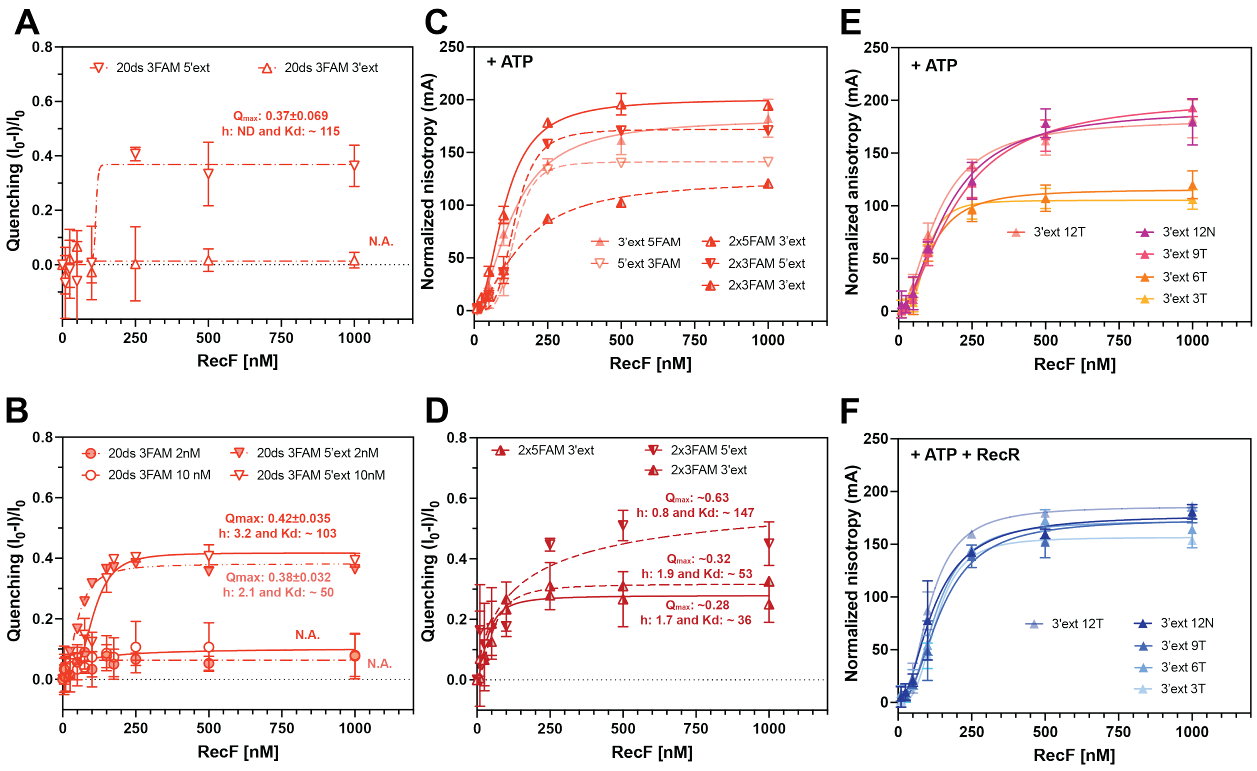

Supporting figure 4: Quenching intensity observed for 20mers with ssDNA extensions and the effect of the length of the 3' extension on RecF binding.

The RecF binding reaction were carried out (**A**, **B**, **C**, **D**, **E)** in the presence of 3 mM ATP (shades of red) or (**F)** in the presence 3 mM ATP and 5 μM RecR (shades of blue). The values plotted represent the mean of reactions carried out in triplicate and the standard deviation obtained for each RecF concentration. The curves represent the Hill slope fit binding model obtained for each condition. In the case of the quenching intensity, plots **A**, **B**, and **D**, the parameters obtained from the Hill slope fit are also indicated. **A.** The quenching intensity observed for 20mer dsDNA FAM labeled on a 3' end, with the complementary strand having either a 5' or 3' extension. **B** The quenching intensity observed for 20mer dsDNA FAM labeled on a 3' end, with the complementary strand having a 5' extension was repeated with either 2nM or 10nM of DNA probe and compared to the quenching intensity observed for the RecF binding to 2nM or 10 nM of 3FAM dsDNA lacking an extension. **C** The RecF binding to dual labeled DNAs possessing additional 5'or 3’extensions was tested in presence of 3 mM ATP and compared to the binding observed for the corresponding singly labeled DNA previously presented in Figure 5 and represented here with 50% transparency. Binding to 2x5FAM

3'ext (right half top triangle), 2x3FAM 5'ext (left half down triangle) and 2x3FAM 3'ext (left half down triangle) was tested. Curves of 5FAM labeled DNAs are shown as solid lines while curves of 3FAM labeled DNAs are shown as dashed lines. **D** The quenching intensity observed upon binding to dual labeled DNA with additional extensions was plotted, the symbols were identical as panel **C**. **E** and **F** RecF binding to dsDNA (5FAM) with a 3′ extension varying in length (9, 6 or 3T) or in composition (12N) was tested in presence of 3 mM ATP (**E,** red shades) and in the presence of 3mM ATP and 5 μM RecR (**F**, blue shades) and compared to values obtained in Figure 5 with the 20mer ds harboring a 3′ extension (12T) represented with 50% transparency here.

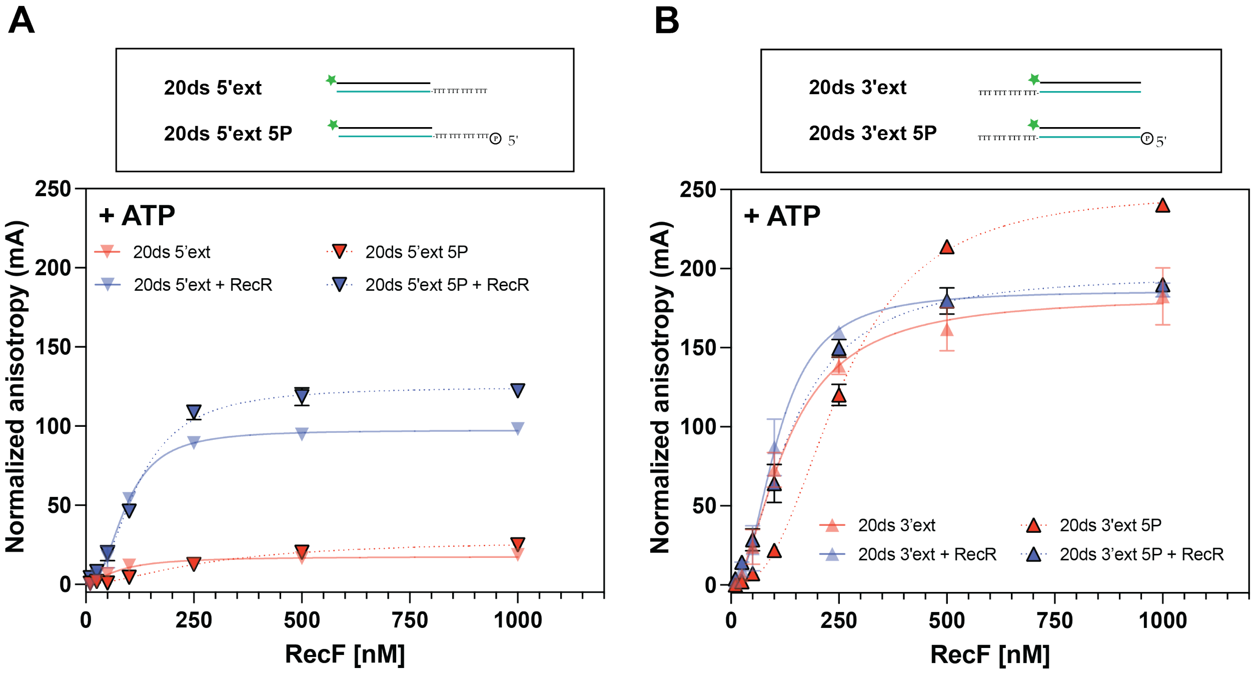

Supporting figure 5: Effects of 5′ phosphoryl groups

Binding is shown to a number of DNA substrates with comparisons shown for substrates with (dotted lines) and without a 5′ phosphoryl group (solid line), in presence of 3 mM ATP (red) or 3mM ATP and 5μM RecR (blue). Values obtained in Figure 5 with the 20mer ds harboring a 3′ or 5′ extension (12T) represented with 50% transparency here. In panel A, the 5′ phosphoryl group is placed on the end of the 5′ ssDNA extension. In panel B, the 5′ phosphoryl group is at the end of the duplex portion of the DNA substrate.

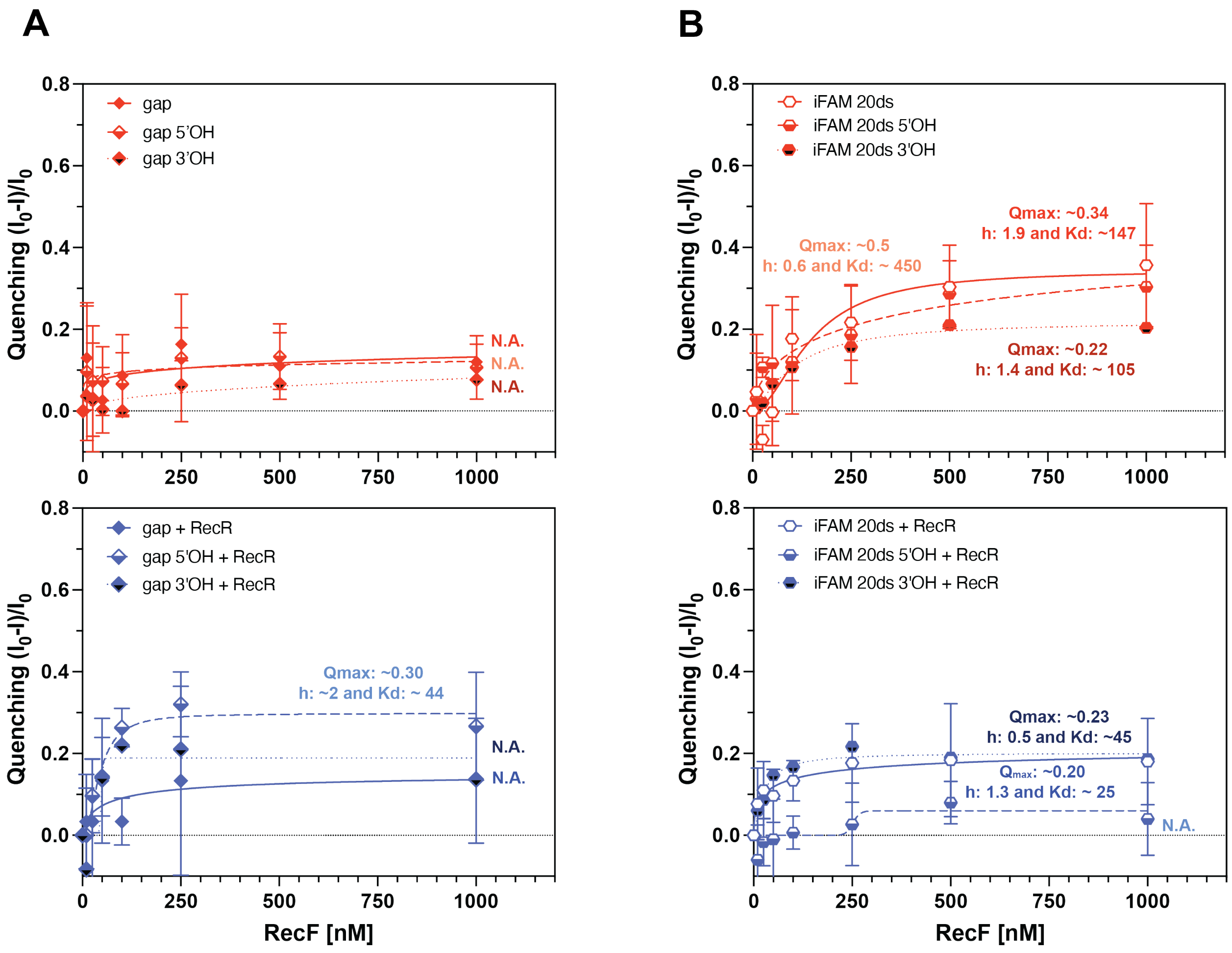

Supporting figure 6: Quenching intensity observed for gapped DNA substrate

The quenching intensity observed upon binding to gapped DNA probes, with or without an additional 5' or 3' unpaired ssDNA extension. RecF binding experiments were carried out in the presence of 3 mM ATP (top panels, red) or in presence of 3 mM ATP and 5 μM RecR (bottom panel, blue). The values plotted represent the mean of reactions carried out in triplicate and the standard deviation obtained for each RecF concentration The curves represent the Hill slope fit binding model obtained for each condition. If applicable, the parameters derived from the Hill slope fit are indicated. **A** The quenching intensity observed for a single strand gap formed between two hairpins lacking a ssDNA extension, or with a 5 or 3' unpaired ssDNA extension. **B** The quenching intensity observed for a circular gapped DNA with 20 bp of dsDNA, with or without unpaired ssDNA extensions.

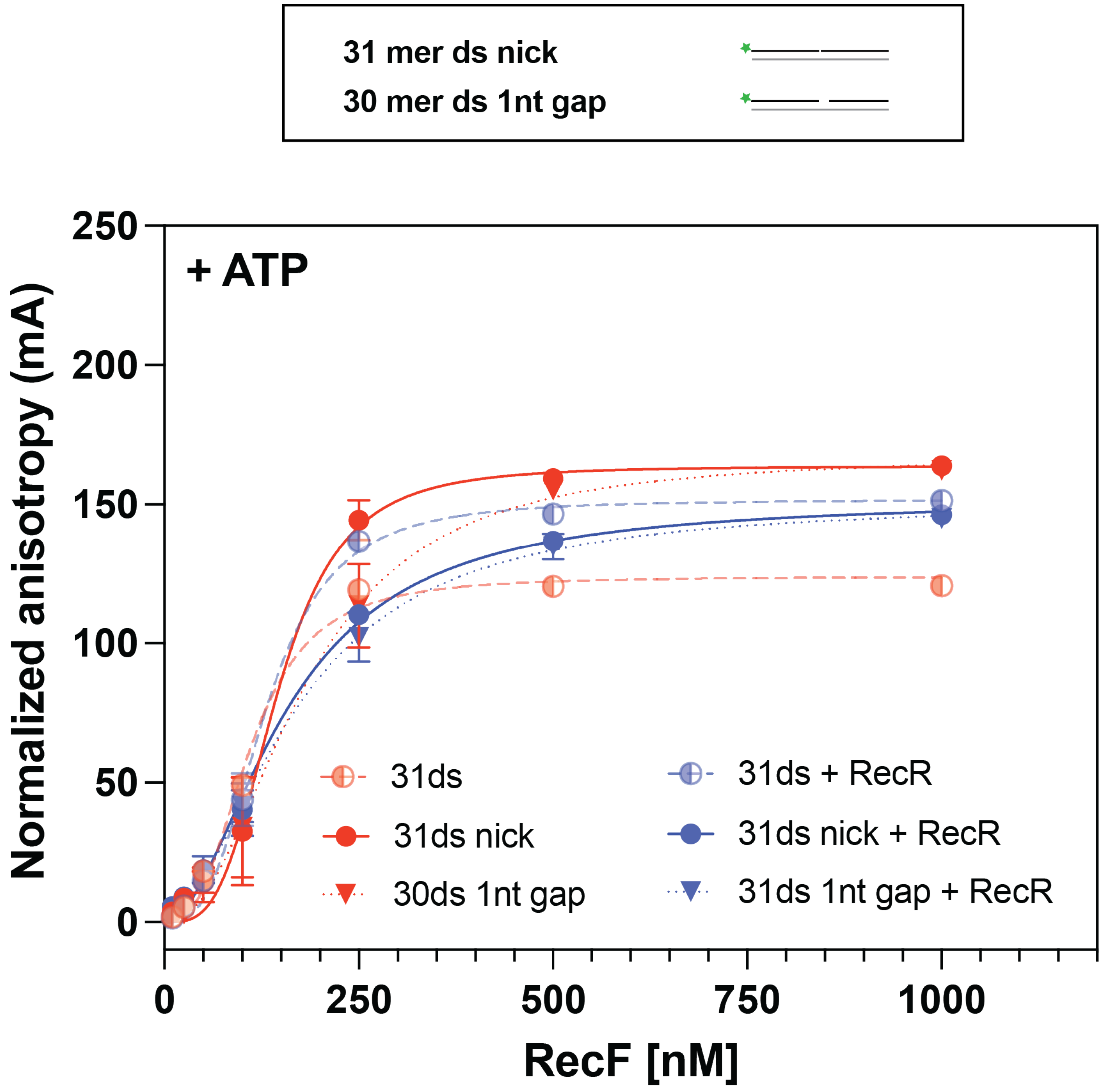

Supporting figure 7: Binding to substrates presenting a nick or a 1nt gap

Binding to substrates of 31 mer composed of 31 ds with a nick (solid line) or 30bp with gap in the middle (dashed line) was tested in presence of 3 mM ATP (red) or 3mM ATP and 5μM RecR (blue) and compared to the binding observed for the corresponding 31mer dsDNA labeled DNA previously presented in Figure 2 and represented here with 50% transparency.
